# Preventable Hospitalization and Primary Healthcare Visits Among Hypertensive Patients in Makkah City

**DOI:** 10.3390/healthcare13233039

**Published:** 2025-11-25

**Authors:** Turky Arbaein

**Affiliations:** Department of Health Administration and Hospitals, College of Public Health and Health Informatics, Umm Al-Qura University, Makkah 24382, Saudi Arabia; tiarbaein@uqu.edu.sa

**Keywords:** hypertension, primary healthcare, ambulatory care sensitive conditions, preventable hospitalization, and Saudi Arabia

## Abstract

**Background:** Hypertension-preventable hospitalizations are used internationally as indicators of primary healthcare centers (PHCs) performance. The Ministry of Health (MoH) in Saudi Arabia has recently implemented several programs to strengthen PHCs and reduce avoidable admissions. This study aimed to observe recent trends in healthcare utilization among hypertensive patients in Makkah City. **Methods:** A retrospective comparative time-trend analysis was conducted using aggregated monthly counts from individual-level records collected by the Makkah Health Cluster between January 2023 and May 2024. Data from 43 PHCs and all public hospitals were analyzed using segmented Poisson regression based on the AHRQ Prevention Quality Indicator #07 (PQI-07) for hypertension. Models included demographic covariates (age, sex, nationality) and a dummy variable to adjust for the Eid holiday period. Rates were calculated per 100,000 adults. **Results:** A total of 42,743 hypertension-related encounters were identified, including 2895 preventable hospitalizations and 39,848 PHC visits. Monthly analyses showed a 5.7% decline in preventable hospitalizations (*IRR* = 0.943; *p* < 0.001) and a 1.5% increase in PHC visits (*IRR* = 1.015; *p* < 0.001). Reductions were most pronounced among adults ≥ 65 years. Non-Saudis were significantly less likely to attend PHCs, reflecting public-sector eligibility differences. **Conclusions:** After controlling for Eid-related seasonal variation, the results indicate a favorable shift toward outpatient hypertension management in Makkah City. These findings align with Saudi Vision 2030 objectives of strengthening PHC and reducing preventable hospital burden. Further multi-regional studies integrating private-sector data are warranted to confirm national trends.

## 1. Introduction

Hypertension, or high blood pressure, is classified as a major chronic disease and a modifiable risk factor for cardiovascular disease. Globally, hypertension affects approximately 1.7 billion (≈30%) of the world’s adult population [1]. It is estimated to rise in the coming years due to the aging of the population and increased exposure to urban lifestyles [1]. Hypertension is therefore a major public-health challenge contributing to high morbidity and mortality worldwide [2]. In Saudi Arabia, as with many other countries undergoing rapid lifestyle transitions, hypertension has emerged as a significant public health concern [3]. The rate of hypertension in Saudi Arabia has risen from 4.1 million (25%) to 5 million (29%) in 2019 [4]. The Western region of Saudi Arabia, where Makkah is located, has a notably higher prevalence of hypertension (≈36%) compared to the national average [5]. According to a study conducted in Makkah in 2024, less frequent visits to PHCs is linked to poorer blood pressure control and a higher risk of complications among hypertensive patients [6]. This highlights a significant public health concern leading to complications. These complications include ischemic heart disease, stroke, heart failure, and kidney damage [7]. Thus, improving access to outpatient healthcare settings is crucial for better managing hypertension.

In response, the Ministry of Health (MoH) has prioritized the strengthening of Primary Healthcare Centers (PHCs). It launched several initiatives such as the Modern Healthcare Model, Family Medicine Expansion, Chronic Disease Management Programs, Medication-Refill and Teleconsultation Services, and Home Blood-Pressure Monitoring [8,9,10,11]. These programs enhance early detection, follow-up, and adherence among hypertensive patients. As a result, they help reduce avoidable hospital admissions. When hypertension is poorly controlled, patients may experience acute complications such as heart failure or hypertensive crises that require hospitalization [12]. Many of these admissions, however, are considered preventable if timely and effective outpatient care is provided [8,9].

Preventable hospitalizations are admissions for conditions that could have been effectively managed in outpatient settings, such as PHCs [13]. To quantify such events, the Agency for Healthcare Research and Quality (AHRQ) developed the Prevention Quality Indicators (PQIs) [14]. These indicators identify hospitalizations that could be avoided through appropriate ambulatory care [14]. The indicator PQI #07 (Hypertension Admission) is specifically used to measure hypertension-preventable hospitalizations [14]. Hospitalizations are more resource-intensive and expensive than outpatient care settings. Hence, hypertension-preventable hospitalization rates represent an important research focus for improving healthcare efficiency in Makkah, Saudi Arabia. One of the primary objectives of the MoH in Saudi Arabia is to enhance access to PHCs, thereby reducing the load on hospitals [15]. One of the indicators of improved access to healthcare among hypertensive patients is the utilization rate of outpatient settings or PHCs [16]. More PHCs utilization is associated with lower rates of preventable hospitalization [17]. Conversely, lower visits to PHCs indicate limited access to needed care and, consequently, higher rates of preventable hospitalizations [17]. Therefore, examining the trend of both PHCs visits and preventable hospitalizations among patients with hypertension offers a valuable tool. It helps in assessing the efficacy and the effectiveness of the PHCs interventions in mitigating hypertension-related complications that require hospitalization and inpatient management [5].

Several national studies have examined the prevalence and risk factors of hypertension in Saudi Arabia, and many others have focused on the utilization of PHCs and hospital access in Makkah City [5,13,18,19,20]. However, to the best of our knowledge, none have explored temporal relationships between hypertension visits to PHCs and hypertension-preventable hospitalizations at the local level, particularly in Makkah City. It remains unclear whether recent investments in PHCs have translated into measurable reductions in hypertension-preventable hospitalization.

This study therefore aimed to observe monthly trends and comparative patterns of hypertension-preventable hospitalizations and hypertension visits to PHCs in Makkah City from January 2023 to May 2024.

The study hypothesized that:

1. Hypertension visits to PHCs increased over the study period, reflecting enhanced access and management capacity;
2. Hypertension-preventable hospitalizations decreased correspondingly, indicating improved outpatient effectiveness; and
3. The magnitude of these trends varied by age, sex, and nationality.

By addressing these aims, the study contributes local empirical evidence to assess the effectiveness of recent PHC-strengthening efforts within the broader goals of Saudi Vision 2030, which emphasizes prevention, accessibility, and healthcare system efficiency.

## 2. Materials and Methods

### 2.1. Study Design

This is a retrospective comparative time-trend analysis to observe hypertension-preventable hospitalization and hypertension visits to PHCs in Makkah City, Saudi Arabia, between January 2023 and May 2024.

Individual-level patient records from primary healthcare centers (PHCs) and public hospitals were aggregated into monthly counts to evaluate temporal changes in hypertension-preventable hospitalizations and hypertension visits to PHCs.

The study employed descriptive statistics to summarize the categorical and continuous variables. Also, a segmented Poisson regression model was used to estimate monthly rate changes and the influence of demographic and seasonal factors on healthcare utilization. Moreover, the study incorporated Eid holiday dummy variable into all models to adjust for predictable declines in healthcare activity during Eid holidays. This design enabled comparative assessment of trends across healthcare levels while accounting for demographic and seasonal variations.

### 2.2. Data Sources

The study utilized two datasets from the Ministry of Health (MoH) via the Makkah Health Cluster:

1. PHC dataset: Included all hypertension visits in 43 PHCs in Makkah City during the study period. Each record contained patient age, sex, nationality, visit date, and ICD-10 diagnosis codes.
2. Hospital dataset: Included all public hospital admissions within the same geographic area and period. Records included patient demographics, admission and discharge dates, and the principal diagnosis (ICD-10).

Both datasets were de-identified before analysis, and the study received ethical approval from the Ministry of Health Institutional Review Board (IRB approval number: 24-85 E).

### 2.3. Identifying Hypertension Cases in the Datasets

The study identified Hypertension cases according to the Agency for Healthcare Research and Quality (AHRQ) Prevention Quality Indicator #07 (PQI-07).

- Hypertension preventable hospitalizations were defined as inpatient admissions where the principal diagnosis matched PQI-07 ICD-10 codes for hypertension-related conditions (Table 1) [17]. Following the AHRQ technical specifications, the study excluded all admissions involving transfers or obstetric cases.
- Hypertension visits to PHCs were identified when hypertension appeared as the primary diagnosis in the PHC dataset. The following ICD-10 codes were used: I10 (essential hypertension), I119 (hypertensive heart disease, unspecified), I1310 (hypertensive heart and chronic kidney disease), I129 (hypertensive chronic kidney disease), and I160–I169 (hypertensive crises) (Table 1) [21].

### 2.4. Study Population

The study included adult residents of Makkah City aged 18 years or older, both Saudi and non-Saudi, who had either:

1. A hypertension-visit to a PHC, or
2. A hypertension-preventable hospital admission between January 2023 and May 2024.

All records with missing key demographic variables (age, sex, or nationality) variable were excluded. Also, the study excluded all records that lacked or if hypertension was listed as a secondary diagnosis.

The study included a total of 42,743 hypertension-related encounters, comprising 2895 hypertension preventable hospitalizations and 39,848 hypertension visits to PHCs.

### 2.5. Variables and Definitions

- Dependent variables:
  ○ Monthly count of hypertension- preventable hospitalizations, and
  ○ Monthly count of hypertension-visits to PHCs visits.
- Independent variables:
  ○ Time (months): Continuous variable representing January 2023 to May 2024.
  ○ Age group: 19–34, 35–49, 50–64, ≥65 years.
  ○ Sex: Male, Female.
  ○ Nationality: Saudi, Non-Saudi.
  ○ Eid holiday: Dummy variable coded *1* for months overlapping Eid (April 2023 and April 2024) and *0* otherwise.

The study calculated the rates per 100,000 adults, using mid-year population estimates for Makkah City provided by the General Authority for Statistics.

### 2.6. Data Management and Cleaning

The study merged both datasets using unique, de-identified patient identifiers. Duplicate entries were removed by matching identifiers and visit/admission dates. 

Also, Quality checks ensured that diagnostic codes corresponded to valid hypertension classifications per PQI-07 specifications.

### 2.7. Statistical Analysis

The study used descriptive statistics to summarize categorical variables as counts and percentages.

To analyze time trends and identify predictors of healthcare utilization, the study employed segmented Poisson regression models to monthly aggregated counts for both outcomes: hypertension preventable hospitalizations and hypertension visits to PHCs. The models included time (months) as the main continuous variable and Eid holiday as a control for seasonal variation. Additional covariates included age group, sex, and nationality.

Results were reported as Incidence Rate Ratios (IRRs) with 95% Confidence Intervals (CIs), representing the relative monthly change in rates. Model diagnostics assessed overdispersion, autocorrelation, and goodness-of-fit, confirming the suitability of the Poisson specification. All statistical analyses were performed using Stata/IC version 17.0 (StataCorp, College Station, TX, USA), with a significance level of *p* < 0.05 [22].

### 2.8. Ethical Considerations

The study adhered to the ethical standards of the Ministry of Health Institutional Review Board (IRB No. 24-85 E) and the Declaration of Helsinki. All data were anonymized before analysis, and no patient-identifiable information was used or disclosed.

## 3. Results

### 3.1. Demographic Characteristics:

The study reported a total of 42,743 records during the study period, comprising 2895 hypertension preventable hospitalizations and 39,848 hypertension visits to PHC (Table 2). Table 2 shows the majority of cases were reported among adults aged 50–64 years (39.4% of hospitalizations; 46.8% of PHC visits) and those aged 65 years or older (33.3% and 27.3%, respectively). Together, individuals aged 50 years and above accounted for nearly three-quarters of all encounters (73% of hospitalizations; 74% of PHC visits) (Table 2). In terms of gender, females represented a slightly higher proportion of hospitalizations (53.8%) compared with males (46.2%). In contrast, PHC visits were distributed almost equally between males (49.7%) and females (50.3%). Regarding nationality, Saudi nationals comprised the majority of both preventable hospitalizations (84.7%) and PHC visits (73.6%). However, non-Saudi residents demonstrated a proportionally higher share of PHC visits (26.4%) relative to their hospital admissions (15.3%). The overall rate of hypertension-preventable hospitalizations was 228.7 per 100,000 adults in Makkah, while the rate of hypertension visits to PHC was 3147.9 per 100,000 adults in Makkah during the study period.

### 3.2. Predictors of Hypertension Preventable Hospitalizations:

Table 3 summarizes the multivariable Poisson regression results examining factors associated with hypertension-preventable hospitalizations. Time (measured in months) was a significant predictor (*IRR* = 0.943; *p* < 0.001), indicating a 5.7% monthly decrease in hypertension-related preventable hospitalizations across the study period. Age showed a graded inverse association: patients aged 50–64 years had a 1.5% decrease (*IRR =* 0.985; *p* = 0.008), and those aged ≥ 65 years had a 3.2% decrease (*IRR* = 0.968; *p* < 0.001) compared with the 19–34 reference group. Females exhibited a slightly higher rate of hospitalization (*IRR* = 1.013; *p* < 0.001), though the absolute difference was modest (+1.3%). Saudi nationals had a 1.2% higher hospitalization rate compared with non-Saudis (*IRR* = 1.012; *p =* 0.001). The Eid holiday period was strongly associated with a sharp reduction in hospitalizations (*IRR* = 0.258; *p* < 0.001), reflecting a 74.2% decline due to decreased service utilization during holidays. The study controlled the effects of the Eid period in the model to ensure that observed time trends were independent of seasonal holiday fluctuations.

### 3.3. Predictors of Hypertension Visits to PHC:

Table 4 shows the regression results for factors influencing hypertension visits to PHCs. Time (months) was again a significant predictor (*IRR* = 1.015; *p* < 0.001), corresponding to a 1.5% monthly increase in PHC visits. Age groups did not show statistically significant differences in PHC utilization compared with younger adults. Females had a marginally higher likelihood of PHC visits (*IRR* = 1.002; *p* < 0.001) compared to male. Saudi nationals were slightly more likely than non-Saudis to visit PHCs (*IRR* = 1.005; *p* < 0.001). The Eid period again showed a notable decline in PHC activity (IRR = 0.735; *p* < 0.001), indicating a 26.5% reduction in visits during holidays.

**Figure 1:**
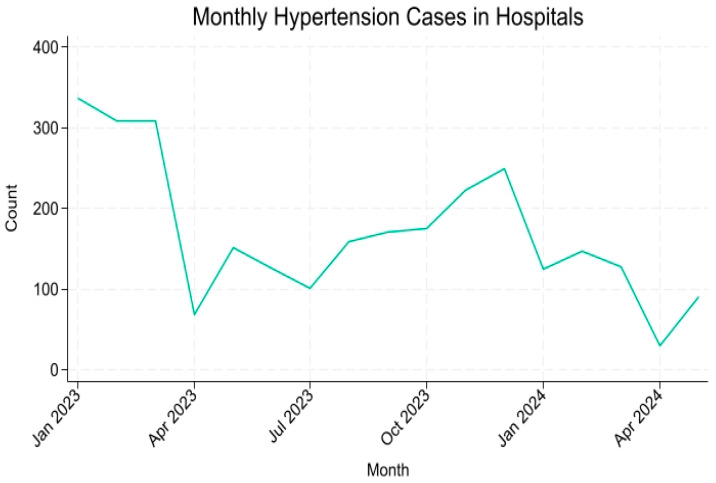
Monthly Trends in Hypertension-Preventable Hospitalizations in Makkah (January 2023–May 2024).

Figure 1 illustrates the monthly trend in hypertension-preventable hospitalizations from January 2023 to May 2024. The figure shows a steady downward trend in hospitalizations over time. A sharp dip in April 2023 and April 2024, corresponding to the Eid holidays.

**Figure 2:**
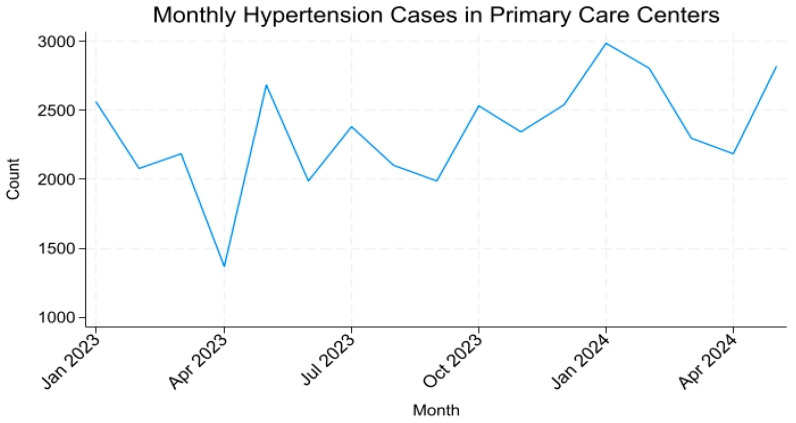
Monthly Trends in Hypertension Visits to Primary Healthcare Centers in Makkah (January 2023–May 2024).

Figure 2 shows the trend in hypertension visits to PHCs visits across the same period. In contrast to hospitalizations, PHC visits demonstrated a consistent upward trend with a noticeable drop during the same holiday period.

Together, Figure 1 and Figure 2 highlight a diverging pattern, declining hospitalizations alongside rising PHC visits, suggesting a favorable shift in hypertension management within Makkah’s healthcare system.

## 4. Discussion

This study is among the first in Saudi Arabia to observe comparative time trends in hypertension-preventable hospitalizations and hypertension visits to primary PHCs visits in Makkah City, Saudi Arabia. Using aggregated data from the Makkah Health Cluster and applying segmented Poisson regression models adjusted for Eid periods, the results demonstrate a clear divergent pattern: preventable hospitalizations for hypertension steadily declined, whereas PHC visits for hypertension increased between January 2023 and May 2024. These findings suggest that the utilization of primary care services for hypertension management has improved during the study period, reflecting ongoing health-system transformation in alignment with Saudi Vision 2030 objectives [8,9,10].

### 4.1. Interpretation of Key Findings

The observed 5.7% monthly decrease in preventable hospitalizations and 1.5% monthly increase in PHC visits indicate a favorable shift in healthcare utilization. Importantly, because the regression models controlled for Eid holidays, this trend cannot be attributed solely to temporary service closures or reduced access during holiday periods. Instead, it likely reflects enhanced PHC capacity and continuity of care achieved through the MoH initiatives such as the Modern Healthcare Model and the Family Medicine expansion program [8,9].

Age was an important factor influencing hypertension healthcare utilization. In preventable hospitalization, the decline was most pronounced among adults aged 65 years and older, who experienced a 3.2% monthly reduction compared with younger age groups. This finding suggests that older adults may have benefited most from such as strengthened chronic-disease follow-up, medication-refill systems, and home-monitoring programs implemented across PHCs. This aligns with the MoH initiatives targeting elderly care and continuity of services. This finding supports previous studies showing that increased access to primary care reduces hospitalization risk among elderly populations [23,24].

Gender differences were minimal, consistent with reports that hypertension management outcomes are becoming increasingly comparable between men and women in the Kingdom [25]. Nationality, however, emerged as a relevant determinant: non-Saudi residents were significantly less likely to utilize PHC services. This disparity likely reflects eligibility constraints within the public PHC system. Many non-Saudis receive care through the private sector via employer-based insurance [26,27]. Prior national surveys also underscored the importance of including private-sector data in future monitoring to avoid underestimating hypertension management coverage among expatriate populations.

The downward trend in hypertension-preventable hospitalizations parallels findings with international studies linking expanded PHC access to reductions in ambulatory-care-sensitive admissions [23,28]. Similarly, the study observed a steady increase in hypertension visits to PHCs. This aligns with other outcomes from PHC reforms in other middle-income settings, where chronic disease programs have improved early management and adherence [10,28].

In the Saudi context, earlier national analyses described a noticeable increasing in hypertension admission rates [5,13,18]. This is suggesting that recent reforms may now be translating into measurable system-level benefits. The inclusion of PQI #07 as a performance indicator strengthens these results by aligning with internationally recognized definitions of preventable hospitalizations [14,17]. Moreover, by using Eid-adjusted segmented time-trend modeling, this study overcomes limitations in previous cross-sectional work that did not account for seasonal variation or short-term service interruptions.

### 4.2. Policy and Practical Implications

From a health-policy standpoint, consistent monitoring of PQIs can serve as a local performance metric for the Modern Healthcare Model. It also enables regional clusters to evaluate whether resource allocation aligns with reduced hospital burden and improved outpatient management. Such data-driven monitoring also contributes to national goals under Vision 2030, emphasizing efficiency, prevention, and equitable access [8,27].

These findings provide evidence-based insight into the evolving balance between hospital and PHC utilization for hypertension in Makkah City. The results support continued investment in PHC infrastructure, workforce, and digital integration to sustain the observed decline in hypertension-preventable hospitalizations. Targeted outreach to non-Saudi residents, potentially through public–private partnerships, may help reduce disparities in PHC use [26,29]. Overall, the study findings underline the importance of accessible, high-quality PHCs as a foundation of chronic disease management. They also show its role in reducing hospital burden and improving overall population health.

### 4.3. Strengths and Limitations

This study’s major strength lies in its use of comprehensive, individual-level datasets aggregated across both hospitals and PHCs within a Makkah healthcare cluster. The application of segmented Poisson regression with seasonal adjustment provides a robust and transparent approach to measuring temporal changes.

Nevertheless, several limitations should be acknowledged. First, the study period of 17 months is relatively short, capturing only early signals rather than long-term effects. Second, the study included only public-sector data. Many non-Saudis as well as Saudis receive care in private facilities, which were not captured in this study. This may bias the utilization estimates. Third, although the models controlled for Eid-related seasonality, other unmeasured factors such as coding practices, health-campaign timing, or patient behavioral change could influence the observed trends. Finally, the ecological design precludes causal inference; results indicate association, not direct cause-and-effect relationships, between PHC use and hospitalization rates.

### 4.4. Future Directions

Future research should extend the observation period, incorporate private-sector datasets, and explore cost-effectiveness analyses of PHC interventions. Linking clinical outcomes such as blood-pressure control or medication adherence to utilization trends would also clarify how strengthening PHC translates into patient-level benefits.

### 4.5. Conclusions

This study observed a significant reduction in hypertension-related preventable hospitalizations alongside an increase in hypertension visits to PHC in Makkah City between 2023 and 2024. These findings suggest progress toward a more prevention-oriented healthcare system. Also, it highlights the potential of PHC investments to alleviate hospital burden. Continued monitoring of PQIs and inclusion of private-sector data will be essential to sustain and generalize these improvements across Saudi Arabia.

## Figures and Tables

**Table 1:** PQI#07 Hypertension—Preventable Hospitalizations.

ICD-10 Code	Diagnosis Name	Category	Notes
I10	Essential (primary) hypertension	Essential Hypertension	Common, uncomplicated high blood pressure
I119	Hypertensive heart disease, unspecified	Complication: Heart Disease	No mention of heart failure or kidney disease
I1310	Hypertensive heart & chronic kidney disease, stage 1–4 or unspecified, without heart failure	Complication: Heart + Kidney	Both systems involved, no heart failure
I129	Hypertensive chronic kidney disease, unspecified stage	Complication: Kidney Disease	Kidney involvement without heart disease
I160	Hypertensive urgency	Hypertensive Crisis (Urgency)	Severe BP without organ damage
I161	Hypertensive emergency	Hypertensive Crisis (Emergency)	Severe BP with organ damage
I169	Hypertensive crisis, unspecified	Hypertensive Crisis (Unspecified)	No specific classification of crisis type

**Table 2:** Demographic Characteristics of the Studied Population (*n* = 42,743).

Variables		Hypertension Preventable Hospitalization (*n* = 2895)	Hypertension PHC Visits (*n* = 39,848)
***Rates of Hypertension Cases (Per 100,000 adults)***	From 1/2023 5/2024	228.7	3147.9
***Age group***	19–34	168 (5.8%)	1393 (3.5%)
	35–49	618 (21.5%)	8914 (22.4%)
	50–64	1136 (39.4%)	18,569 (46.8%)
	65 or above	959 (33.3%)	10,832 (27.3%)
***Gender***	Male	1337 (46.2%)	19,813 (49.7%)
	Female	1558 (53.8%)	20,035 (50.3%)
***Nationality***	Non-Saudi	443 (15.3%)	10,537 (26.4%)
	Saudi	2452 (84.7%)	29,311 (73.6%)

**Table 3:** Multivariable Poisson Regression Analysis of Factors Influencing Hypertension-Related Preventable Hospitalizations, Makkah City (January 2023–May 2024).

Hypertension-Preventable Hospitalizations				
Factors	IRR	% Change	[95% CI Lower, Upper]	*p*-Value
**Time in Months (from 1/2023 to 5/2024)**	0.943	−5.7%	[0.943, 0.944]	**0.000**
**Age group (Ref: 19–34)**				
35–49	0.993	−0.7%	[0.982, 1.004]	0.219
50–64	0.985	−1.5%	[0.975, 0.996]	0.008
65 or above	0.968	−3.2%	[0.957, 0.979]	**0.000**
**Sex (Ref: Male)**				
Female	1.013	1.3%	[1.008, 1.018]	**0.000**
**Nationality (Ref: Non-Saudi)**				
Saudi	1.012	1.2%	[1.005, 1.019]	**0.001**
**(Ref: Non-Eid)**				
Eid holidays	0.258	−74.2%	[0.251, 0.265]	**0.000**

**Table 4:** Multivariable Poisson Regression Analysis of Factors Influencing Hypertension Visits to Primary Healthcare Centers, Makkah City (January 2023–May 2024).

Hypertension Visits to Primary Healthcare Centers				
Factors	IRR	% Change	[95% CI Lower, Upper]	*p*-Value
**Time in Months (from 1/2023 to 5/2024)**	1.015	1.5%	[1.015, 1.015]	**0.000**
**Age group (Ref: 19–34)**				
35–49	0.999	−0.1%	[0.998, 1.001]	0.281
50–64	0.999	−0.1%	[0.998, 1.001]	0.324
65 or above	1.001	0.1%	[1, 1.002]	0.172
**Sex (Ref: Male)**				
Female	1.002	0.2%	[1.001, 1.002]	**0.000**
**Nationality (Ref: Non-Saudi)**				
Saudi	1.005	0.5%	[1.004, 1.005]	**0.000**
**(Ref: Non-Eid)**				
Eid holidays	0.735	−26.5%	[0.734, 0.735]	**0.000**

## Data Availability

Access to the dataset is limited to authorized personnel within the Ministry of Health and those who have obtained official permission from the Ministry.
